# An efficient *Potato virus X* -based microRNA silencing in *Nicotiana benthamiana*

**DOI:** 10.1038/srep20573

**Published:** 2016-02-03

**Authors:** Jinping Zhao, Qingtao Liu, Pu Hu, Qi Jia, Na Liu, Kangquan Yin, Ye Cheng, Fei Yan, Jianping Chen, Yule Liu

**Affiliations:** 1 Center for Plant Biology, MOE Key Laboratory of Bioinformatics, School of Life Sciences, Tsinghua University, Beijing 100084, China; 2 The State Key Laboratory Breeding Base for Sustainable control of Pest and Disease, Hangzhou, 310021, China; 3 Key Laboratory of Biotechnology in Plant Protection of MOA of China and Zhejiang Province, Institute of Virology and Biotechnology, Zhejiang Academy of Agricultural Sciences, Hangzhou 310021, China

## Abstract

Plant microRNAs (miRNAs) play pivotal roles in many biological processes. Although many miRNAs have been identified in various plant species, the functions of these miRNAs remain largely unknown due to the shortage of effective genetic tools to block their functional activity. Recently, miRNA target mimic (TM) technologies have been applied to perturb the activity of specific endogenous miRNA or miRNA families. We previously reported that *Tobacco rattle virus* (TRV)-based TM expression can successfully mediate virus-based miRNA silencing/suppression (VbMS) in plants. In this study, we show the *Potato virus X* (PVX)-based TM expression causes strong miRNA silencing in *Nicotiana benthamiana*. The PVX-based expression of short tandem target mimic (STTMs) against miR165/166 and 159 caused the corresponding phenotype in all infected plants. Thus, a PVX-based VbMS is a powerful method to study miRNA function and may be useful for high-throughput investigation of miRNA function in *N. benthamiana*.

Plant microRNAs (miRNAs) are a class of endogenous non-coding small RNAs with 20–24 nucleotides^1^,^2^. Plant miRNAs mainly act as negative regulators of gene expression at the post-transcription level by triggering endonuclease cleavage or by promoting translation repression of target mRNAs^3^. They therefore play essential roles in various biological processes including growth and development^4^,^5^, metabolism^6^,^7^, hormone signaling^8^,^9^, and responses to biotic^10^,^11^,^12^,^13^ and abiotic^14^,^15^,^16^ stresses. The functions of plant miRNAs can be investigated by enhancing their activity through ectopic- or over- expression of the miRNA gene^9^,^17^ or by blocking miRNA function through expression of a miRNA-resistant target that has uncleavable silent mutations in the mRNA sequence^18^. Despite great progress in predicting and identifying miRNAs in many plants, characterization of their biological roles has largely lagged behind, especially in non-model plant species. This is for two reasons: on the one hand, traditional loss-of-function approaches are difficult to apply due to the extensive genetic redundancy of miRNA genes in plant genomes^19^,^20^; on the other hand, the use of miRNA resistant targets can only partially reveal the function of those miRNAs that regulate multiple targets^21^,^22^,^23^.

miRNA function is naturally regulated by target mimics (TMs). The TM molecules have a mismatch bulge at the position corresponding to nucleotides 10–11 of the relative miRNA, the cleavage site of target mRNAs, thus blocking the cleavage of target mRNAs by miRNA-Argonaute proteins^24^,^25^. Artificially designed TM transcripts based on Arabidopsis *INDUCED BY PHOSPHATE STARVATION 1* (*IPS1*) are highly complementary to specific miRNAs but contain extra nucleotides that can form a mismatching loop at the cleavage site in the miRNA-TM duplex, thus preserving TMs from cleavage and sequestrating miRNA functions^25^. A short tandem target mimic (STTM), consisting of two TMs separated by a short linker sequence, suppressed miRNA activity more efficiently than the regular TM in transgenic plants^26^,^27^,^28^. Thus miRNA TM technology provides an effective method to manipulate the activity of endogenous miRNAs and dissect their function(s)^27^,^29^,^30^.

We previously developed a plant virus-based miRNA expression system, in which a *Tobacco rattle virus* (TRV)-based vector was used to express artificial TMs and cause virus-based miRNA silencing/suppression (VbMS)^31^,^32^. Several research groups have developed VbMS systems based on different plant viruses, including *Cotton leaf crumple virus* (CLCrV)^33^ and *Cucumber mosaic virus* (CMV)^34^,^35^ and have demonstrated good miRNA suppression. VbMS enables a prompt elucidation of miRNA function and has proved to be effective in *Nicotiana benthamiana*^31^,^34^, *N. tabacum*^34^, *Solanum lycopersicum*^31^,^34^, *Arabiopsis*^32^,^35^ and *Gossypium hirsutum*^33^. However, the efficiency of VbMS caused by currently available TRV vectors is not strong enough and weak phenotypes may be overlooked^31^,^32^.

The precise mode of action of TMs is unknown, but TM molecules induce small RNA degradation by pairing with target miRNA molecules^27^ and the levels of TM transcripts inversely correlate with those of the targeted miRNAs^25^,^27^,^36^. Thus viral vectors which provide higher accumulations of TM transcripts could cause higher VbMS efficiency. PVX derived vectors can be used to mediate high expression of foreign genes^37^,^38^ and so might be modified to deliver improved VbMS.

In this study, we found that PVX-based VbMS causes very efficient silencing of miRNAs in *N. benthamiana* and so may be a useful tool for high-throughput investigation of miRNA function in plants.

## Results

### Development of PVX-based VbMS vector

**Figure 1:**
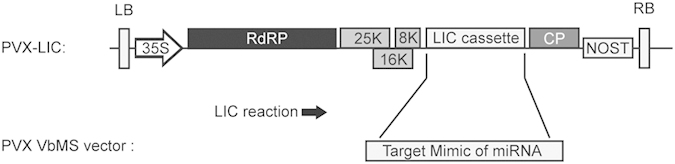
Diagram of the Potato Virus X based PVX-LIC vector for VbMS. The PVX-LIC vector was generated by introducing the LIC cassette into a T-DNA PVX vector. Artificially designed TM sequences can be cloned into PVX-LIC via the LIC reaction and expressed under the control of the CP subgenomic promoter. LB: T-DNA left border, RB: T-DNA right border, 35S: *Cauliflower mosaic virus* 35S promoter, NOSt: nopaline synthase terminator, RdRP: RNA dependent RNA polymerase, 25K: PVX 25K protein, 16K: PVX 16K protein, 8K: PVX 8K protein, CP: coat protein.

To perform high-throughput cloning of TMs, we generated a modified PVX vector PVX-LIC (Fig. 1) by cloning a ligation independent cloning (LIC) cassette into pSPDK658^39^. We showed previously that STTM-mediated VbMS suppressed miRNA more strongly than regular *IPS1*-based VbMS^31^. Therefore, we used the PVX-based vector to express STTMs under the control of the coat protein (CP) sub-genomic promoter (sgP) to inhibit miRNA function in this study (Fig. 1). The designed STTM sequences can be cloned into PVX-LIC by the high throughput LIC method.

### PVX-based VbMS of miR165/166

**Figure 2:**
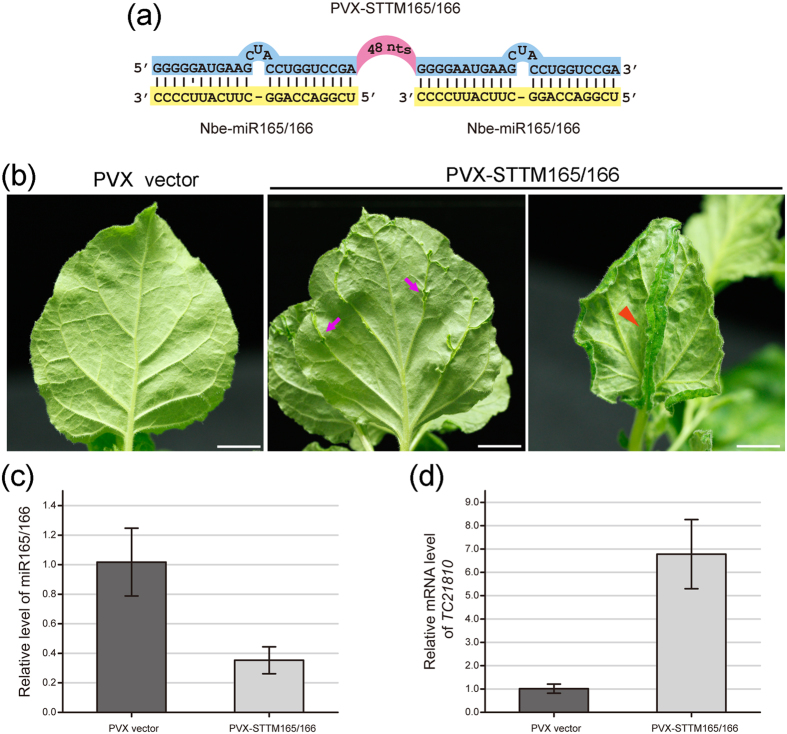
Suppression of miR165/166 by PVX-STTM165/166 in *Nicotiana benthamiana.* (**a**) Scheme of the base pairing pattern of STTM165/166 and miR165/166. -: denotes no nucleotide at this position. 48 nts: the 48-nt long stem-loop linker. (**b**) Leaf phenotypes of plants inoculated with PVX-STTM165/166. The abaxial side of leaves from the PVX control and PVX-STTM165/166 plants were photographed 15 days post infiltration (dpi). Magenta arrows denote the ectopically generated leaf tissues in the leaf veins, orange arrow heads indicate the larger leaf lamina formed from the midrib. Bars represent 1 cm. (**c**) Relative miR165/166 levels as measured by stem-loop RT-PCR in plants inoculated with PVX or PVX-STTM159. (**d**) Relative expression levels of the miR165/166 target *HDZIP* like gene *TC21810*. Data are means of 3 independent real-time RT-PCR experiments. Error bars show the standard deviation (±SD).

**Figure 3:**
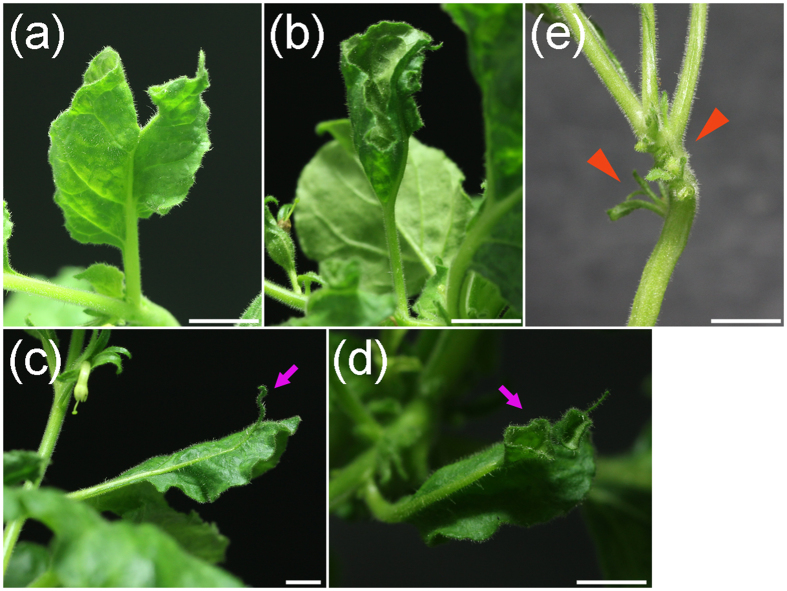
Varied severe phenotypes of miR165/166 blockage by PVX-STTM165/166 in *Nicotiana benthamiana.* (**a**) Abnormal bilateral symmetric leaf with double layered lamina split by the mid vein. (**b**) Cup-shaped leaf with adaxial surface on the outside and abaxial surface inside. (**c**) Midrib growing out from the lamina and forming rod like terminus (arrow). (**d**) Trumpet-shaped leaves generated along the midrib (arrow). (**e**) Ectopic axillary meristems at the vicinity of leaf insertion sites; arrow heads indicate outgrowth of leave tissues from the stem surface. Bars represent 1 cm.

In *N. benthamiana*, miR165/166 is predicted to target the *homeodomain-leucine zipper* (*HDZip*) gene family. In an earlier study, TRV-based expression of STTM against Nbe-miR165/166 (STTM165/166) led to reduced apical dominance, and only in extreme cases caused generation of an ectopic leaf from the midrib^31^. PVX-based expression of STTM165/166 (Fig. 2a) caused a strong silencing phenotype (Figs 2 and 3) with pleiotropic developmental defects. The most general defect was ectopic leaf outgrowths from veins on the leaf abaxial surface (Fig. 2b). A leaf lamina appeared on the back side of the leaf rib with opposite symmetry to the normal leaf (Fig. 2b). In some cases, alteration of the adaxial-abaxial organization led to the formation of abnormal bilateral symmetric leaves with a double layered lamina split by the mid vein (Fig. 3a) and cup-shaped leaves (Fig. 3b), reflecting an alteration of the leaf primordia caused by blockage of miR165/166^40^. Occasionally, the midrib grew out from the lamina (Fig. 3c) and in certain extreme cases, trumpet-shaped leaves were generated along the rib of the adaxial side of some leaves in addition to the terminally extended mid vein (Fig. 3d). At the nodes, leaves grew out of the stem from ectopically generated axillary meristems near leaf insertion sites (Fig. 3e), comparable to the strong phenotypes of the miR165/166 resistant *PHAVOLUTA* (*NsPHAV*) in *Nicotiana sylvestris*^40^.

All PVX-STTM165/166 infiltrated plants displayed obvious leaf developmental defects, implicating a great efficiency of VbMS. Real time RT-PCR analysis verified that the mRNA levels of the predicted Nbe-miR165/166 target *TC21810*, a *HD-Zip* gene, were elevated greatly in PVX-STTM165/166 infiltrated plants, (Fig. 2c) whereas Nbe-miR165/166 levels were highly reduced (Fig. 2d).

### PVX-based VbMS of miR159

In Arabidopsis, miR159 targets a set of transcription factor genes containing the MYB domain that are involved in vegetative development and flowering^41^,^42^. Deep sequencing data show that miR159 is conserved in *N. benthamiana*^43^,^44^,^45^ and it is predicted that Nbe-miRNA159 cleaves several *MYB* like transcription factor genes (http://plantgrn.noble.org/psRNATarget/, *N. benthamiana* genome version 1.0.1). The exact biological function of miR159 in *N. benthamiana* development has not been studied but it is reported to have a role in the pathogenesis of severe CMV strains^34^,^35^.

**Figure 4:**
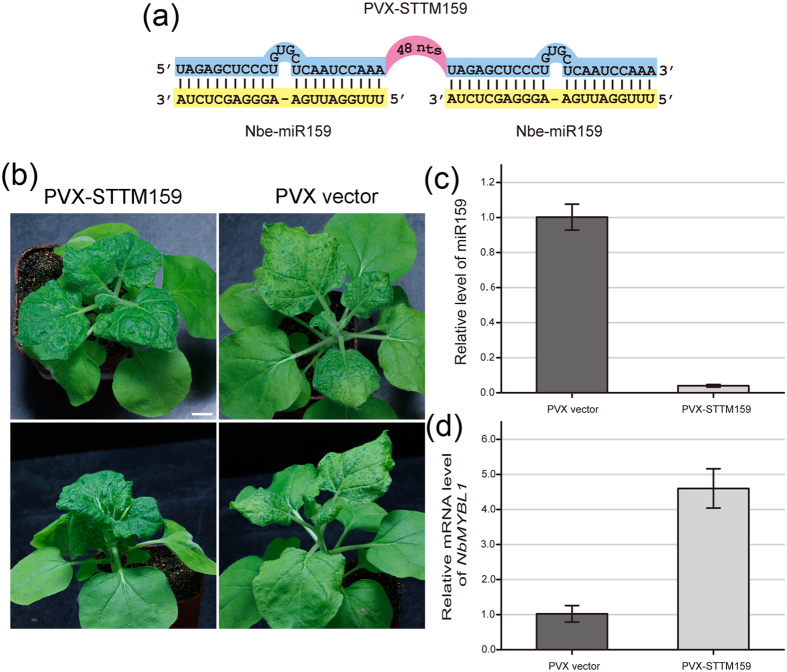
PVX-based VbMS of miR159 by PVX-STTM159 in *Nicotiana benthamiana.* (**a**) Diagrammatic representation of PVX-STTM159. -: denotes no nucleotide at this position. 48 nts: the 48-nt long stem-loop linker. (**b**) Plants infiltrated with PVX control or PVX-STTM159 photographed at 20 dpi. Top row, top view; bottom row, side view. Bar represents 1 cm. (**c**) Relative miR159 levels as measured by stem-loop RT-PCR in plants inoculated with PVX or PVX-STTM159. (**d**) Relative mRNA levels of miR159 target *NbMYBL1* in the PVX control and PVX-STTM159 plants. Values are means ± SD from 3 independent experiments.

In our experiments, PVX-STTM159 inoculated plants were stunted and compact with downward-curled leaves, darker green pigmentation, smaller leaf area and shorter petioles than PVX control plants (Fig. 4a), indicating that functional blockage of miR159 triggers serious leaf morphogenesis defects. In addition, the internodes of PVX-STTM159 plants were shorter and the leaves were clustered together at the shoot apex (Fig. 4b). Compared to the PVX control plants, the mRNA levels of the miR159 target, *MYB*-like transcription factor (*NbMYBL1*, *N. benthamiana* genome version 1.0.1, Niben101Scf01383g07027.1), were increased in PVX-STTM159 plants while the miR159 levels decreased greatly (Fig. 4c,d). These results indicate that the PVX VbMS method can inhibit different endogenous miRNAs and provide an indication of miRNA/target mRNA function.

## Discussion

In this study, we have shown that the modified PVX vector can be used to express TM molecules and successfully inhibit the function of several endogenous miRNAs in *N. benthamiana*. There was a very high efficiency of miRNA silencing in the plants. All PVX-STTM165/166 infiltrated plants exhibited ectopic leaf outgrowths, similar to those observed in the gain-of-function HD-ZIP III *phv1* mutants in *N. sylvestris*^40^ and in plants with CMV-based VbMS of miR165/166^34^. By comparison, in *N. benthamiana* plants infected with TRV containing STTM-165/166 (TRV-STTM165/166), ectopic leaf outgrowths on the leaf middle vein were observed only in certain extremes^31^. All PVX-STTM159 *N. benthamiana* plants had a leaf developmental defect (Fig. 4), and resembled the phenotypes induced by CMV-based VbMS of miR159 as well as a severe CMV strain Fny-CMV. However, we did not observed any leaf phenotype in TRV-STTM159 plants. These results suggest that the PVX based VbMS is highly effective in blocking the function of targeted miRNAs in *N. benthamiana*.

PVX (in this study) or CMV^34^ seems to have a higher efficiency of VbMS than our previously reported TRV system for the miRNAs expressed in leaves of *N. benthamiana*. It is possible that TRV is a better VIGS vector and that VIGS may cause a lower level of viral RNA containing TM. Since TRV has a wide host range and also infects the meristems of plants, whereas PVX and CMV do not, TRV-based VbMS is still very useful for dissecting the function of many miRNAs expressed in the meristems and in some important host plants.

In the previous report, the success of miRNA silencing depended on the expression levels of the TM transcripts. Highly expressed TMs may trigger more effective silencing effects on targeted miRNAs and greater enhancement of miRNA target mRNA levels. Meanwhile, different designed TMs may have differing effectiveness in plant cells^25^,^27^,^36^.

Manipulation of the sequence composition of the TM molecule, by inserting 1–3 nucleotides to produce central mismatches between the cleavage site or at other recognition sites, provides subtle regulation of target miRNA inactivation^36^,^46^. It has been reported that no single design can guarantee the most dramatic silencing of different miRNA families^29^. However, it appears that the most efficient TMs are obtained quite empirically; the most important factor is to avoid the formation of another new cleavage site as previous reported^29^.

In addition to *N. benthamiana*, PVX-mediated gene expression has been reported to function in *N. clevelandii*, *N. tabacum*, *Solanum tuberosum* and *S. lycopersicum* in either local or systemic tissues^47^,^48^. The high levels of foreign gene products synthesized and the long term expression produced by PVX makes the system suitable for miRNA TM expression throughout the duration of plant development^30^,^49^. Thus PVX can be utilized as a general vector for various plants. As PVX can tolerate extra larger insertions and repetitive regions with high homology^37^,^50^, it may be feasible to insert multiple TM segments into the PVX vector to promote expression of heterogenic TM molecules. The PVX VbMS may provide an excellent system for engineering different miRNA families involved in related or unrelated processes in plants. Therefore, the modified PVX-based VbMS described in this report has great potential for the characterization of functions of miRNAs.

## Materials and Methods

### Plant Growth

*N. benthamiana* plants were grown in a growth room at 24 °C with a 16 hr/8 hr light/dark photoperiod cycle, under white light at 100 μM m^−2^ s^−1^. Four week old plants with 7–8 leaves were used for PVX VbMS infiltration.

### TM molecular design

The 3~4 nt insertions in STTM molecules were designed empirically at the site corresponding to nts 10–11 of the targeted miRNA. All PCR reactions for cloning were amplified by using the synthesized 48 nt oligonucleotide as template with EasyPfu polymerase (Transgene). All primers used for cloning are listed in Supplementary Table S1.

### Plasmid Construction

PVX-LIC was generated by inserting the LIC cassette containing the *ccdB* and chloramphenicol-resistant genes into the PVX T-DNA vector^39^ by replacing the original multiple cloning site, and was maintained and propagated in *E*. *coli* strain DB3.1. Before LIC cloning, PVX-LIC was digested with *SmaI* and treated with T4 DNA polymerase in the presence of dTTP (0.5 mM) and DTT (1 mM) for 30 min at 37 °C to produce the 14 nt sticky 5′ end. After the polymerase was inactivated at 75 °C for 20 min, the sticky-ended PVX-LIC vector was purified by phenol extraction and ethanol precipitation. TM PCR products were treated with T4 DNA polymerase in the presence of dATP (0.5 mM) and DTT (1 mM), followed by a 75 °C inactivation step and purified by ethanol precipitation. For LIC cloning, equal volumes of PVX-LIC and PCR product treated with T4 DNA polymerase were mixed and incubated at 37 °C for 30 min, and then transformed into *E*. *coli* strain DH5α. The resulting constructs were verified by sequencing and transformed to *Agrobacterium tumefaciens* strain GV3101 (or GV2260).

### Agrobacteria infiltration

Agrobacterium strain GV3101 (or GV2260) carrying the PVX derivatives was grown at 28 °C overnight, then harvested by centrifugation at 5,000 g and re-suspended in infiltration buffer (10 mM MES, 10 mM MgCl_2_ and 200 μM acetosyringone) to a final OD_600_ of 1.0. The suspensions were incubated at 25 °C for 2.5 ~ 4 hrs and infiltrated into leaves of *N. benthamiana* with needleless syringes. Each experiment was performed three times with at least 6 plants for each construct. Symptom development was monitored starting from 10 days after infiltration. Plants exhibiting phenotypes were then photographed and tissues were collected for subsequent analyses.

### RNA extraction and Real time RT-PCR analysis

Total RNA was isolated using TRNzol-A^+^Reagent (Tiangen Biotech), treated with DNase I (Sigma Aldrich) to eliminate genomic DNA and converted into cDNA using the QuantScript reverse transcription Kit (Tiangen Biotech). Real-time PCR primers were designed with Primer Express 3.0. Real-time PCR was performed using SYBR Green PCR Master Mix (Life technologies) with sequence-specific primers. Stem loop RT-PCR was performed to analyze mature miRNA levels as described previously^51^. *N. benthamiana eukaryotic Initiation Factor-4A* (*eIF4A*) was used as an internal control and each assay was replicated at least three times. The expression data illustrated by the quantification cycle (Cq) were collected, statistically processed by the *ΔΔ*Cq algorithm and plotted using Origin 2015 software. The primers used in RNA analysis are shown in Supplemental Table S1.

## Additional Information

**Accession codes:** *NbAP2L1* (Genbank Accession number CK287095); *eIF4a* (Dana-Farber Cancer Institute *N. benthamiana* Gene Index TC19454).

**How to cite this article**: Zhao, J. *et al.* An efficient *Potato virus* X -based microRNA silencing in *Nicotiana benthamiana*. *Sci. Rep.* **6**, 20573; doi: 10.1038/srep20573 (2016).

## Supplementary Material

Supplementary Information
