# Supplementary material for: An efficient Potato virus X -based microRNA silencing in Nicotiana benthamiana
Source: Sci Rep. 2016 Feb 3;6:20573. doi: 10.1038/srep20573 (PMC4738334; doi:10.1038/srep20573)
Supplement: Supplementary Information [file srep20573-s1.pdf]

Efficient *Potato virus X* -based microRNA silencing in *Nicotiana benthamiana*

Jinping Zhao [a, b, #,*], Qingtao Liu [a, #], Pu Hu [a], Qi Jia [a], Na Liu [a], Kangquan Yin [a], Ye Cheng [b], Fei Yan [b], Jianping Chen [b], Yule Liu [a, *]

[a] Center for Plant Biology, MOE Key Laboratory of Bioinformatics, School of Life Sciences, Tsinghua University, Beijing 100084, China

[b] The State Key Laboratory Breeding Base for Sustainable control of Pest and Disease, China; Key Laboratory of Biotechnology in Plant Protection of MOA of China and Zhejiang Province, Institute of Virology and Biotechnology, Zhejiang Academy of Agricultural Sciences, Hangzhou 310021, China

[#] These authors contributed equally to this work

*** Corresponding authors:**

Name	Yule Liu
Telephone	+86-10-62794013
Fax	+86-10-62794013
E-mail	yuleliu@mail.tsinghua.edu.cn

Name	Jinping Zhao
Telephone	+86-571-86416069
Fax	+86-571-8616069
E-mail	jinpingzhao@sina.cn

Supplementary Table S1. Primers Used in Vector Construction and PCR Analysis.

**Primer ID**	**Primer Sequence (5’-3’)**	**Brief Description**
Stem-loop 165/166 [c]	GTCTCCTCTGGTGCAGGGTCCGAGGTATTC GCACCAGAGGAGACgGGGGAATGAAG	For stem-loop RT of miR165/166
miR165/166 FP [b]	CGGCTCGGACCAGGCTT	For miR165/166 Real time PCR
Stem miR165/166 RP	GTGCAGGGTCCGAGGT	For miR165/166 Real time PCR
STTM 165/166 FP [a]	CgACgACAAgACCgTGGGGGATGAAGctaCCT GGTCCGAgttgttgttgttatgg	For cloning STTM165/166
STTM165/166 RP [a]	gAggAgAagAgCCgTTCGGACCAGGtagCTTCA TTCCCCattcttcttctttagacca	For cloning STTM165/166
miR165/166 target FP	ATCCGCCAAGGGATGCTAGT	For *TC21810* Real time PCR
miR165/166 target RP	TCGGCTCTAGACCAACCAGG	For *TC21810* Real time PCR
Stem-loop miR159 [c]	GTCTCCTCTGGTGCAGGGTCCGAGGTATTC GCACCAGAGGAGACGAGCTCCCTT	For stem-loop RT of nbt-miR159
miR159 FP [b]	GCGGGCGTTTGGATTGAAG	For miR159 Real time PCR
Stem miR159 RP	GTGCAGGGTCCGAGGT	For miR159 Real time PCR
STTM 159 FP [a]	CgACgACAAgACCgTTAGAGCTCCCTgtgcTC AATCCAAAgttgttgttgttatgg	For cloning STTM159
STTM 159 RP [a]	gAggAgAagAgCCgTTTTGGATTGAgcacAGGG AGCTCTAattcttcttctttagacca	For cloning STTM159
miR159 target FP	CATCACCCATCATTCTCCGGT	For *NbMYBL1* Real time PCR
miR159 target RP	GACCACTGTTCCGAGGTGAC	For *NbMYBL1* Real time PCR
eIF4A FP	GCTTTGGTCTTGGCACCTACTC	For *eIF4A* Real time PCR
eIF4A RP	TGCTCGCATGACCTTTTCAA	For *eIF4A* Real time PCR
48 nts	GTTGTTGTTGTTATGGTCTAATTTAAATATGG TCTAAAGAAGAAGAAT	As STTM template

Supplementary Table S1. Primers Used in Vector Construction and PCR

[a], STTM mimic sequence is underlined; [b], MiRNA sequence is underlined; [c], Reverse-complement sequence of miRNA is underlined.
